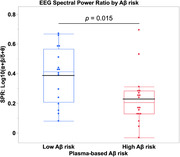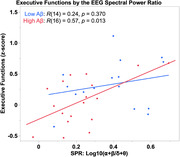# The EEG spectral power ratio as a cognitively relevant marker of preclinical AD

**DOI:** 10.1002/alz.089481

**Published:** 2025-01-09

**Authors:** Peter J. Fried, Christie Morse, Andrew Northrop, Rushali Patel, Eliza L McKinney, Keya Shah, Danylo Ferreira Cabral, Stephanie S. Buss, Christopher Benwell, Recep Ozdemir, Mouhsin Shafi

**Affiliations:** ^1^ Berenson‐Allen Center for Noninvasive Brain Stimulation, Beth Israel Deaconess Medical Center and Harvard Medical School, Boston, MA USA; ^2^ Division of Psychology, School of Social Sciences, University of Dundee, Dundee UK

## Abstract

**Background:**

Alzheimer’s disease (AD) is associated with cerebral slowing on electroencephalography (EEG), or a shift in oscillatory power from higher to lower frequencies. This change can be captured as the spectral power ratio (SPR) of alpha and beta over delta and theta: (α+β)/(δ+θ). Prior studies have shown that compared to cognitively unimpaired older adults, the SPR was lower in persons with a clinical diagnosis of dementia due to probable AD and those with a biomarker‐confirmed diagnosis of amnestic mild cognitive impairment (aMCI) due to AD, and a lower SPR was associated with worse executive functions scores. Moreover, the SPR was unaffected in individuals who had amyloid‐negative aMCI, suggesting cerebral slowing may result from AD‐associated pathology. We sought to test this hypothesis in a cognitively unimpaired population using plasma Aβ42/Aβ40 levels as a surrogate biomarker of brain amyloid.

**Methods:**

Eyes‐closed resting‐state EEG, neuropsychological testing, and plasma Aβ42/Aβ40 levels were obtained from 34 adults (18 women, aged 65‐89 years), as part of an ongoing study. EEG data were cleaned and analyzed using custom scripts built around EEGLAB and TESA toolboxes in Matlab. Aβ42/Aβ40 <0.1 was used as a cutoff to indicate a high risk of brain amyloid (Rissman et al., 2023, Alzheimer’s and Dementia, DOI: 10.1002/alz.13542). An executive functions composite score was created by averaging age‐normed z‐scores from the digit symbol substitution, digit span backwards, semantic fluency, verbal fluency, and trail making tests.

**Results:**

A Shapiro‐Wilk test indicated SPR values were not normally distributed, so data were log10‐transformed. 18 participants (53%) were classified as “high‐risk of brain amyloid.” An independent‐samples t‐test showed the SPR_log10_ (mean ±SD) was lower in this group (0.23 ±0.2) than those with “low‐risk of brain amyloid” (0.39 ±0.2), which was significant, t(32) = ‐2.6, p = 0.015. Pearson’s correlation coefficients showed that executive functions were significantly correlated with SPR_log10_ in the high‐risk group, R(16) = 0.57, p = 0.013, but not in the low‐risk group, R(14) = 0.24, p = 0.370.

**Conclusions:**

Cerebral slowing is an early feature of amyloid‐associated pathophysiology. The EEG SPR (α+β)/(δ+θ) is a sensitive and cognitively relevant marker of preclinical AD.